# Toxic intracellular anabolite levels of tenofovir and didanosine causing a steep CD4-cell decline

**DOI:** 10.1186/1758-2652-13-S4-P95

**Published:** 2010-11-08

**Authors:** E de Jong, ME Haverkort, R ter Heine, RS Jansen, JH Beijnen, MA van Agtmael

**Affiliations:** 1 VU University Medical Center, Department of Internal Medicine, Amsterdam, Netherlands; 2 Academic Medical Center, Department of Infectious Diseases, Amsterdam, Netherlands; 3 Slotervaart Hospital, Department of Pharmacy & Pharmacology, Amsterdam, Netherlands

## Introduction

HIV-protease inhibitors may increase tenofovir plasma AUC by 22-37%. Whether this affects tenofovir-diphosphate (TFV-DP) intracellular levels, especially in the presence of didanosine, which is also eliminated through active tubular secretion, is unclear.

## Case report

A 52-year-old HIV-1 positive Caucasian male started zidovudine (AZT), lamivudine, nelfinavir in 1999 at a CD4-cell count of 210/µL. In July 2007 treatment was switched because of viral blips to atazanavir, ritonavir, tenofovir, emtricitabine and didanosine (250 mg). Within one year his CD4-cell count declined from 1140 to 140/µL despite complete virological suppression [1]. Renal clearance (Cockgroft-Gault) decreased from 86 to 74 mL/min and renal phosphate threshold to 0.24 mmol/L (n=0.8-1.35), indicative of proximal tubular dysfunction. There was 8 kg weight loss, his serum glucose and lactate were elevated.

In addition, following the ART-switch a thrombocytosis (1355x10^9^/L) was noticed. After exclusion of other causes, essential thrombocythemia was diagnosed and hydroxyurea started. Thrombocytes were elevated before initiation of ART (427x10^9^/L) and before therapy switch (659x10^9^/L), suggesting AZT-related bone marrow suppression may have prevented a further increase in platelet count in the preceding years.

Suspecting NRTI-related mitochondrial and tubular dysfunction, we measured intracellular ddA-TP (didanosine) and TFV-DP (tenofovir) in PBMCs [2]. TFV-DP was 10xULN (1350 fmol/10^6^ cells) and ddA-TP 21xULN (105 fmol/10^6^ cells). Hydroxyurea may have increased ddA-TP levels, but was used for only 2 weeks. ART was changed to AZT, lamivudine, atazanavir, ritonavir, raltegravir. Two weeks later TFV-DP was still 250 fmol/10^6^ cells, demonstrating an intracellular t½ of approximately 140 hrs and ddA-TP 57.4 fmol/10^6^cells, t½ 385 hrs, but didanosine and tenofovir plasma levels were undetectable. After switch his CD4-cell count increased again from 140 to 340/µL and his platelet count decreased to 725x10^9^/L following re-initiation of AZT.

## Conclusions

Elevated TFV-DP and ddA-TP led to tubular dysfunction and mitochondrial toxicity. Inhibition of purine-nucleoside-phosphorylase by TFV-DP and DNA-polymerase-γ by ddA-TP may have caused the steep CD4-cell decline. We believe interactions between tenofovir, didanosine and atazanavir/ritonavir were responsible for this toxicity.
